# H_3_K_4_ Methylation and Demethylation in Fungal Pathogens: The Epigenetic Toolbox for Survival and Adaptation in the Host

**DOI:** 10.3390/pathogens13121080

**Published:** 2024-12-09

**Authors:** Maruti Nandan Rai, Rikky Rai

**Affiliations:** 1 College of Agricultural, Consumer, and Environmental Sciences (ACES), University of Illinois Urbana-Champaign, Urbana, IL 61801, USA; 2 Department of Botany, University of Allahabad, Prayagraj 211002, Uttar Pradesh, India; rikkyrai@allduniv.ac.in

**Keywords:** fungal pathogens, chromatin, H3K4 methylation, H3K4 demethylation, transcription regulation, stress response, virulence, fungal pathogenesis

## Abstract

Pathogenic fungi represent a diverse group of eukaryotic microorganisms that significantly impact human health and agriculture. In recent years, the role of epigenetic modifications, particularly histone modifications, in fungal pathobiology has emerged as a prominent area of interest. Among these modifications, methylation of histone H3 at lysine-4 (H3K4) has garnered considerable attention for its implications in regulating gene expression associated with diverse cellular processes. A body of literature has uncovered the pivotal roles of H3K4 methylation in multiple biological processes crucial for pathogenic adaptation in a wide range of fungal pathogens of humans and food crops. This review delves into the recent advancements in understanding the impact of H3K4 methylation/demethylation on fungal pathogenesis. We explore the roles of H3K4 methylation in various cellular processes, including fungal morphogenesis and development, genome stability and DNA repair, metabolic adaptation, cell wall maintenance, biofilm formation, antifungal drug resistance, and virulence. We also discuss the conservation of H3K4 methylation regulators and their potential as therapeutic targets to prevent fungal diseases. Collectively, this review underscores the intricate links between H3K4 methylation, fungal pathogenesis, and potential avenues for novel antifungal strategies.

## 1. Introduction

Pathogenic fungi profoundly affect human well-being through various means. Opportunistic human fungal pathogens like *Candida* species, *Cryptococcus neoformans*, *Histoplasma capsulatum*, and *Aspergillus fumigatus* pose a direct and serious threat to the health of immunocompromised individuals by causing superficial to systemic fungal infections [1,2,3]. On the other hand, phytopathogenic fungi, though they primarily infect plants, also indirectly affect humans by causing diseases in food crops of importance, leading to substantial agricultural losses and thereby causing food safety, quality, and availability issues worldwide [4,5,6]. It is estimated that phytopathogenic fungi are responsible for the loss of ~20% of the total crop yield globally and destroy ~125 million tonnes of farm produce every year [5,6].

Upon host invasion, pathogenic fungi face an adverse microenvironment resulting from the primary and secondary host responses and therefore have to be adept at responding well and mounting suitable responses to counteract host surveillance mechanisms to successfully establish infections [7,8,9]. To achieve that, they undertake extensive transcriptional reprogramming [10,11,12]. Chromatin architecture plays a pivotal role in remodeling transcriptional responses and has been observed to be crucial in fungal pathogenesis in multiple fungal pathogens [11,13,14,15,16,17]. The chromatin configuration to express the desired set of genes is usually facilitated by combinatorial post-translational histone modifications [18,19,20]. Different sets of histone modifications (also known as histone code) alter the chromatin accessibility differently, leading to either transcriptionally active or repressive chromatin [13,18,21,22]. Thus, the fine-tuned post-translational histone modification(s) assists the fungal pathogens in better responding to and adapting to the host microenvironment [11,14,17]. Multiple studies have uncovered the roles of histone modifications in various cellular processes that are crucial for pathogenic adaption, such as morphogenesis and development, cell wall and biofilm formation, metabolic adaptation, DNA repair, genome stability, and cell cycle regulation [16,17,23,24,25,26,27]. However, understanding the physiological significance of histone modification in fungal pathogenesis is still an evolving field. A preliminary survey of the published literature on the PubMed database indicates that there are just 135 published articles addressing the roles of epigenetic histone modifications in fungal pathogenesis, indicating that this area warrants further attention from researchers to better understand the molecular mechanisms of fungal pathogenesis. 

### 1.1. Histone Modifications and Chromatin Architecture

Eukaryotic genomes are highly organized nucleoprotein structures commonly referred to as chromatin. Transcriptionally active and inactive chromatins are known as euchromatin and heterochromatin. The basic structural unit of chromatin is the nucleosome, which consists of 146 base pairs of DNA wrapped around a histone octamer complex comprising two units each of H2A, H2B, H3, and H4 histones. Histone H1 acts as a linker between adjacent nucleosomes of chromatin. Histones are among the most highly conserved proteins in eukaryotic organisms and are highly basic in nature, which helps facilitate the DNA–histone interactions [28,29]. Post-translational modifications of the histones modulate chromatin architecture and the accessibility of cellular transcriptional machinery to DNA, thereby constituting the dynamic and intricate epigenetic regulatory system that governs gene expression [20,21]. These modifications, commonly called the ‘histone code’, form a complex combinatorial language being read and interpreted by effector proteins, including various histone readers, writers, and erasers. Different permutations and combinations of ‘types’ and ‘sites’ of these histone modifications lead to various synergistic or antagonistic regulations of gene expression resulting in transcriptional reprogramming in response to diverse environmental stimuli [13,18,20,21]. 

Commonly occurring covalent post-translational modifications of histones include acetylation, methylation, ubiquitination, sumoylation, and phosphorylation of histones’ N- and C-terminal tails [22,30]. Of these, histone acetylation and methylations are the most frequent modifications and have been thoroughly studied in different model systems and organisms. Histone acetylation, a reversible modification of the core histones, is maintained by the opposing actions of two classes of enzymes, histone acetyltransferases (HATs) and histone deacetylases (HDACs) [30,31] Generally, the elevated HDAC activity is associated with tighter binding of histone proteins to DNA, compact nucleosomes, and repressed chromatin states [31]. In contrast, the onset of transcription is usually accompanied by the increased acetylation of histone proteins and relaxed nucleosomes (open chromatin) [32]. Similarly, histone methyltransferases and demethylases maintain histone methylation status at specific lysine residues and contribute to the regulation of chromatin architecture and gene expression [30]. 

### 1.2. Histone Methylation

Histone methylation is a key epigenetic modification wherein methyl group(s) are covalently attached to amino acid side chains of specific lysine or arginine residues of histone proteins. Histone methylation is tightly controlled by two classes of enzymes known as methyltransferases (KMTs) and demethylases (KDMs). KMTs are highly specific for their substrate both regarding lysine residues on the substrate as well as the degree of methylation [33]. Histone methylations are distinct from other modifications in several ways. For example, unlike other histone modifications which primarily modulate chromatin architecture through electrostatic interactions (histone acetylation and phosphorylation), histone methylation does not alter the charge on amino acid residues [33]. Instead, methylated residues act as binding sites for a range of transcription factors and associated regulatory proteins which, in turn, regulate downstream gene expression [34,35]. 

Interestingly, unlike other histone marks which are usually the hallmarks of either active or repressed chromatin status, histone methylation can lead to both active as well as repressed chromatin depending on the type and sites of methylation marks [19,33]. In general, H3K4, H3K36, and H3K79 methylations are considered to be associated with transcriptionally active chromatin, whereas H3K9, H3K27, and H4K20 methylations are thought to represent repressed chromatin [19,33]. This review focuses on H3K4 trimethylation/demethylation and their significance in pathogenic adaptation in human and plant fungal pathogens.

### 1.3. H3K4 Methylation and Demethylation

**Figure 1:**
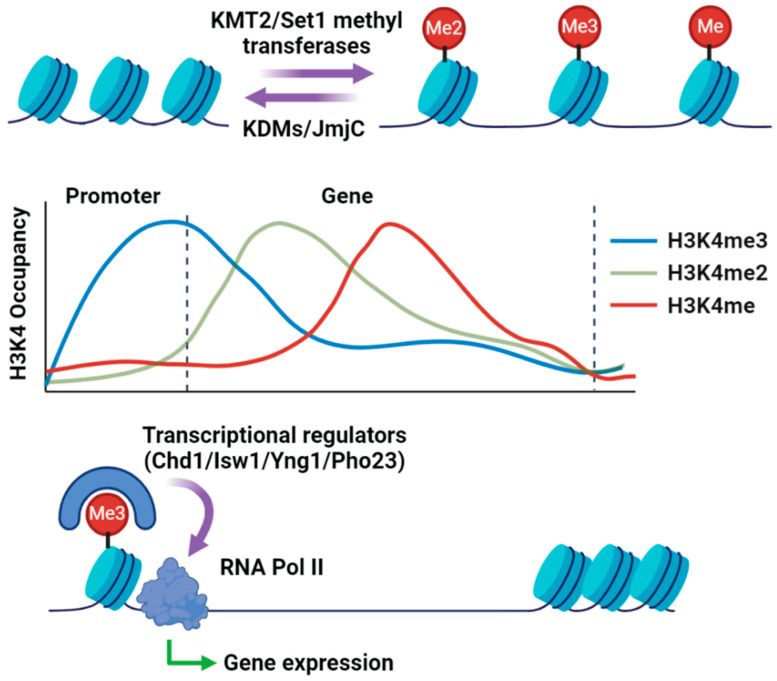
Graphical abstract of histone methylation-mediated transcriptional activation. Created in BioRender. Rai, N. (2024) https://BioRender.com/m31m407, 2 December 2024.

Histone H3K4 methylation is an evolutionary conserved epigenetic modification and is exclusively associated with actively transcribing genes in different organisms, including fungal pathogens [36,37]. H3K4 methylation occurs in three forms: mono-, di-, or trimethylation (Figure 1). Although all three forms are associated with transcriptionally active regions, their positional distribution on the gene body is distinct. While H3K4me3 sites are enriched on the promoter and towards the 5’ region of the gene, H3K4 me2 and H3K4me are concentrated towards the middle and 3’end of the coding regions, respectively (Figure 1) [34]. Histone methylation marks the sites where other transcriptional regulators (e.g., Chd1, Isw1, Yng1, and Pho23) are recruited and facilitate transcription induction (Figure 1) [38,39]. Due to its consistent correlation with active RNA polymerase II occupancy, H3K4me3 was initially thought to be instructive for cellular transcription [40]. However, later, it was found that H3K4 methylation was also associated with chromatin silencing and transcriptional repression functions for certain groups of genes under several stress conditions [40]. It has been proposed that H3K4 methylation serves as transcriptional memory; however, it is noteworthy that H3K4 methylation may occur independently of transcription [41]. Importantly, H3K4 methylation also cross-talks with other histone modifications; for example, H2B ubiquitination has been shown to be pivotal for H3K4 methylation [42,43]. 

H3K4 methylation is facilitated by KMT2 proteins, also known as Set1 methyl transferases, which constitute the subunits of the COMPASS (Complex Proteins Associated with Set1) complex. The COMPASS complex is a catalytically active multimeric complex, consisting of several proteins—Set1, Swd1, Swd2, Swd3, Bre2, Spp1, Sdc1, and Shg1 [44]. The structure and functions of the COMPASS complex are evolutionarily conserved and well studied in model organisms like yeast and drosophila. Set1 acts as a catalytic subunit while other subunits modulate the COMPASS complex’s stability and methyl transferase activity [34]. Set1-mediated H3K4 methylation is pivotal for transcriptional activation and its deletion leads to the downregulation of ~80% of genes in *S. cerevisiae* [43]. Through chromatin organization and gene regulation, H3K4 methylation modulates multiple key cellular processes including DNA repair and genome stability, cell cycle regulation, mRNA splicing, and apoptosis [34]. 

Importantly, despite being strongly associated with active transcription, H3K4 methylation is a dynamic and reversible process. There are two types of histone lysine demethylases, LSD and Jumonji (JmjC). LSDs (also known as KDMs) demethylate di- and mono-methylated H3K4 residues, while JmjC demethylates all three H3K4me3, H3K4me2, and H3K4me residues [45]. In model yeast *S. cerevisiae,* H3K4 demethylation is carried out by a single H3K4 demethylase, Jhd2 [46]. Set1 and Jhd2 colocalize and regulate gene expression by fine-tuning dynamic H3K4 methylation–demethylation levels and recruiting other transcriptional regulators at the site [47]. Because of their critical roles in H3K4 methylation and regulation of various cellular processes, the COMPASS complex, particularly Set1, and Jumonji proteins have garnered the attention of researchers in pathogenic fungi in recent years. A number of research publications have come out investigating the roles of these proteins in fungal pathogens of humans and agricultural crops. The following is a summarized account of studies on the roles of H3K4 methylation in various fungal pathogens.

### 1.4. Roles of H3K4 Methylation and Demethylation in Pathogenic Adaptation in Fungi

(a) Morphogenesis and development

The roles of the COMPASS complex subunits, key players of H3K4 methylation status, in fungal morphogenesis and development are shown to be crucial in both plants as well as human fungal pathogens, including but not limited to *Magnaporthe oryzae*, *Colletotrichum higginsianum*, *Cryptococcus neoformans*, *Penicillium expansum*, and *Fusarium graminearum*. In *M. oryzae,* the causal agent of rice blast disease, MoBre2, MoSpp1, and MoSwd2 constitute integral components of the COMPASS complex and play pivotal roles in fungal development and pathogenicity through the regulation of genes targeted by H3K4me3 modification [27]. The deletion of these genes resulted in impaired hyphal development and pathogenicity [27]. Similarly, in *C. higginsianum*, a brassica anthracnose pathogen, CclA, is required for H3K4 methylation and regulates secondary metabolism, fungal growth, and development [48]. CclA deletion resulted in a marked reduction in appressorium formation and the penetrative ability of the pathogen in an in planta infection model as well as a cellophane membrane model of infection [48]. 

In the human fungal pathogen *C. neoformans,* the causal agent of fungal meningitis, Set1, Bre2, Swd1, and Swd3 form the catalytic core of the COMPASS complex and control the yeast-to-hypha transition, which is needed for fungal dissemination in the host [49]. *Fusarium graminearum*, a plant fungal pathogen, causes fusarium head blight (FHB) in wheat and barley. H3K4 mono-, di-, and tri-methylation in *F. graminearum* is mediated by FgSet1. *ΔFgSet1* mutant exhibits crippled hyphal growth and virulence in vivo [50]. *Penicillium expansum,* a psychrophilic blue mold commonly found in soil, causes blue mold in many fruit plants. The histone methyl transferase PeSet1 regulates host colonization, patulin biosynthesis, and stress responses in *P. expansum,* and its deletion results in severe defects in hyphal growth and conidiation [51]. Another soil fungus, *Metarhizium robertsii*, colonizes the rice rhizosphere and causes diseases in insects including mosquitoes. *M. robertsii* is proposed as a promising bioinsecticide and biofertilizer agent for its cadmium reduction ability in rice and growth promotion [52]. Histone lysine methyltransferase KMT2 in *M. robertsii* (*MrKMT2*) is upregulated upon cuticle induction and plays crucial roles in regulating infection-related morphogenesis and pathogenicity by upregulating the transcription factor MrCre1 via H3K4 trimethylation during mosquito cuticle infection [25]. MrCre1 further regulates the cuticle-induced gene MrHyd4 to modulate infection structure (appressorium) formation and virulence [25]. In another plant pathogen, *Ustilaginoidea virens*, the causal agent of false smut disease in rice, an RNA-seq study revealed that UvKmt2-mediated H3K4me3 plays a pivotal role in transcriptional activation [53]. Further, phenotypic analyses of the *ΔUvkmt2* mutant revealed that *UvKMT2* is necessary for growth, conidiation, secondary spore formation, and virulence in *U. virens* [53]. Together, these studies across diverse fungal pathogens in plants, humans, and insects underscore that H3K4 methylation is critical in fungal morphogenesis and development.

(b) Genome stability and DNA repair

H3K4 methylation is typically associated with actively transcribing genes as well as DNA damage response pathways [54,55]. Genome stability relies on the efficient repair of cellular DNA damage. Transcription–replication conflicts (TRCs) occur in the genomic loci undergoing intensive transcriptional activity during DNA replication resulting in compromised replication fork stability, potentially leading to genetic mutations [56]. H3K4 methylation functionally pairs with the S-phase checkpoint to protect genome stability [55,56]. The interplay between H3K4 methylation and Rad53 kinase contributes to genome stability in response to DNA damage caused by hydroxyurea (HU), particularly in highly expressed genomic regions [56]. Cells that cannot methylate H3K4 residue display a defect in double-strand break (DSB) repair by non-homologous end-joining [55]. Cells lacking H3K4 methyl transferase activity lose viability very rapidly upon encountering DNA damage stress during DNA replication, emphasizing the significant roles of H3K4 methylation in genomic stability [55]. 

A human fungal disease, mucormycosis, caused by *Mucor lusitanicus*, also known as black eye disease, became a serious concern for clinicians and researchers during the recent COVID pandemic, particularly in India [57]. The histone methyl transferase Set1 in *M. lusitanicus* is not only crucial for the growth and sporulation of this fungus but also protects it from DNA-damaging stress [58]. The *M. lusitanicus* mutant lacking Set1 activity displayed elevated sensitivity to SDS, EMS, and UV light, indicating that Set1 is crucial for DNA-damage repair and the pathogenesis of *M. lusitanicus* [58]. *Beauveria bassiana*, an insect-pathogenic fungus, is one of the wide-spectrum fungal insecticides [59]. *DIM5/KMT1* controls fungal pathogenicity and genome stability through the methylation of histone H3K4, H3K9, and H3K36 in *B. bassiana*, and is crucial for the insect-pathogenic lifestyle and genome stability of *B. bassiana* [59]. Although the role of H3K4 methylation in DNA-damage repair, genomic stability, and cell cycle regulation is well documented in a model yeast, *S. cerevisiae*, more studies are warranted in the context of its significance to pathogenic fungi.

(c) Metabolic adaptation

Secondary metabolites play critical roles in the adaptation of both human and plant pathogenic fungi in host environments [60,61,62]. Numerous pieces of evidence indicate that H3K4 methylation actively modulates secondary metabolism in different fungal pathogens [48,50,63,64]. In some pathogenic fungi, such as *F. graminearum*, H3K4 methylation is required for the expression of genes involved in the biosynthesis of the secondary metabolites deoxynivalenol and aurofusarin [50]. In contrast, loss of the histone methyl transferase CclA in the brassica anthracnose pathogen *C. higginsianum* resulted in increased secondary metabolite production and affected growth and development negatively [48]. Similar to *C. higginsianum,* the *ΔcclA* mutant in the human fungal pathogen *Aspergillus fumigatus* displayed poor growth and increased secondary metabolite production [64]. In agreement with this, *Aspergillus nidulans* requires a Jumonji histone demethylase, KdmB, for the induction of secondary metabolism [65]. Although these studies proved the significance of histone H3K4 methylation regulators such as Set1, CclA, and KdmB in fungal secondary metabolism, it remains unclear whether these proteins directly alter H3K4me3 status on their target genes or if it is a consequence of global expression change due to H3K4 methylome alteration. However, given that the secondary metabolite genes are commonly located in telomeric loci, where H3K4 methylation is presumed to play a repressive role in cellular transcription, it is likely to be a result of more direct regulation of secondary metabolite genes. Nonetheless, a clearer understanding of H3K4 methylation-mediated secondary metabolism regulation can ably provide valuable insights into fungal pathogenesis and open up exciting possibilities for discovering novel antifungal compounds.

(d) Cell wall maintenance

In pathogenic fungi, cell wall maintenance and biofilm formation are crucial for adaptation in the host microenvironment. The cell wall acts as a structural barrier protecting fungal cells from the arsenal of the host immune system. It also contributes to biofilm formation, helping pathogens to adhere on host surfaces as well as making them more resilient to host immune responses and antifungal treatment. Genes involved in cell wall biosynthesis and maintenance are modulated by H3K4 methylation. In the model yeast *S. cerevisiae*, Set1-mediated H3K4 methylation is important for the expression of genes associated with the ergosterol biosynthetic pathway, including the rate-limiting enzyme HMG-CoA reductase [24]. *SET1* deletion led to a marked reduction in HMG-CoA reductase protein levels as well as total cellular ergosterol, resulting in increased sensitivity to the antifungal drug brefeldin A (BFA) [24]. In the phytopathogenic fungi *F. graminearum*, the *FgSET1* deletion mutant (*ΔFgSet1*) displayed sensitivity to cell wall-damaging agents [50]. FgSet1 plays an important role in the response to cell wall-damaging agents via negatively regulating the phosphorylation of FgMgv1, a core kinase in the cell wall integrity pathway [50]. *P. expansum* causes blue mold rot in various fruits, and also produces a mycotoxin, patulin, posing a threat to human health [51]. PeSet1 positively regulates key genes in the β-1,3-glucan biosynthetic pathway and thus modulates its cell wall integrity [51]. In another phytopathogen, *Aspergillus flavus,* Set1 is required to survive cell membrane stress [26]. Similarly, in the fungal insect pathogen *Beauveria bassiana*, loss of Set1/Kmt2 activity resulted in elevated sensitivity to cell wall perturbations [59].

(e) Antifungal resistance

Antifungal resistance is often considered to be connected to the ability to respond to cell wall perturbations as some classes of antifungal drugs like echinocandins and azoles target the fungal cell wall and cell membrane. Given that H3K4 methylation is pivotal for the ergosterol biosynthesis pathway in yeast, and multiple reports demonstrate H3K4 methylation’s importance in cell wall integrity in several fungal pathogens, it is likely that it may also affect antifungal drug resistance. However, this topic has not garnered enough attention from researchers so far. Recently, a study by Baker et al., examined the importance of Set1-mediated H3K4 methylation in azole susceptibility in two phylogenetically related organisms, an opportunistic human fungal pathogen, *Candida glabrata*, and the model yeast *S. cerevisiae* [66]. Loss of H3K4 methylation led to elevated azole susceptibility in both *C. glabrata* and *S. cerevisiae* [66]. Interestingly, the increased azole susceptibility in two organisms, lacking *SET1*, involved two distinct mechanisms [66]. While, in *S. cerevisiae*, loss of *SET1* led to downregulation of the efflux pump Pdr5 but not *ERG11* upon azole treatment, loss of *SET1* in *C. glabrata* did not alter the expression of efflux pumps [66]. Instead, *C. glabrata* Set1 was observed to be necessary for the azole-induced expression of all ergosterol biosynthesis genes [66]. Furthermore, this study also reported that H3K4 methylation was induced upon azole treatment in *C. glabrata* and clinical isolates lacking Set1 displayed higher sensitivity towards azole antifungal drugs [66]. In conclusion, this study presented multiple pieces of evidence suggesting that H3K4 methylation is involved in antifungal drug susceptibility in a species-specific manner [66]. It will be interesting to examine if H3K4 methylation modulates drug resistance in other fungal pathogens and to find if H3K4 demethylase *JHD2* corroborates the roles of H3K4 methylation in antifungal drug resistance.

(f) Stress response and virulence

H3K4 methylation-mediated regulation of genes involved in stress response and virulence has been established in multiple human, plant, and insect fungal pathogens. *Candida albicans*, a human opportunistic pathogen, utilizes Set1-mediated H3K4 trimethylation to activate mitochondrial protein genes during host infection to counter oxidative stress generated by the host immune cells [67]. Set1 deletion resulted in elevated ROS accumulation and *Δset1 C. albicans* cells were hypersensitive to external oxidative stress and displayed attenuated virulence in macrophage and murine model of candidiasis [67]. In *Aspergillus* spp., KdmA and KdmB are two Jumonji lysine demethylases, wherein KdmA targets H3K9 and H3K36 residues, while KdmB demethylates H3K4 residues [65,68]. Loss of either KdmA or KdmB in the human pathogen *A. fumigatus* results in decreased expression of both conidia- and mycelia-specific catalases (CatA and Cat1) and increased sensitivity to oxidative stress [69]. Interestingly, the *ΔkdmA* mutant produced more gliotoxin and had no effect on virulence, while *ΔkdmB* displayed lower gliotoxin production and attenuated virulence, suggesting that H3K4 methylation is pivotal in the virulence of *A. fumigatus* [69]. In *Alternaria alternata*, an opportunistic pathogen in ~300 plant species, two H3K4 methyl transferases, AaSet1 and AaSet2, contribute in the adaptation to cell wall and osmotic stresses [70]. The mutants lacking either of these two H3K4 methyl transferases, *∆AaSet1* and *∆AaSet2*, displayed severely impaired pathogenesis [70]. Deletion of PeSet1 in *P. expansum* leads to severe defects in hyphal growth, colonization, patulin biosynthesis, and stress responses [51]. PeSet1 is involved in the regulation of the expression of patulin cluster genes, β-1,3-glucan biosynthesis, and response to oxidative stress [51]. Likewise, many other human and phytopathogenic fungi display marked defects in coping with environmental stresses and pathogenesis upon alteration in H3K4 methylation status [27,49,50,53,58,71]. 

## 2. Discussion and Future Directions

**Figure 2:**
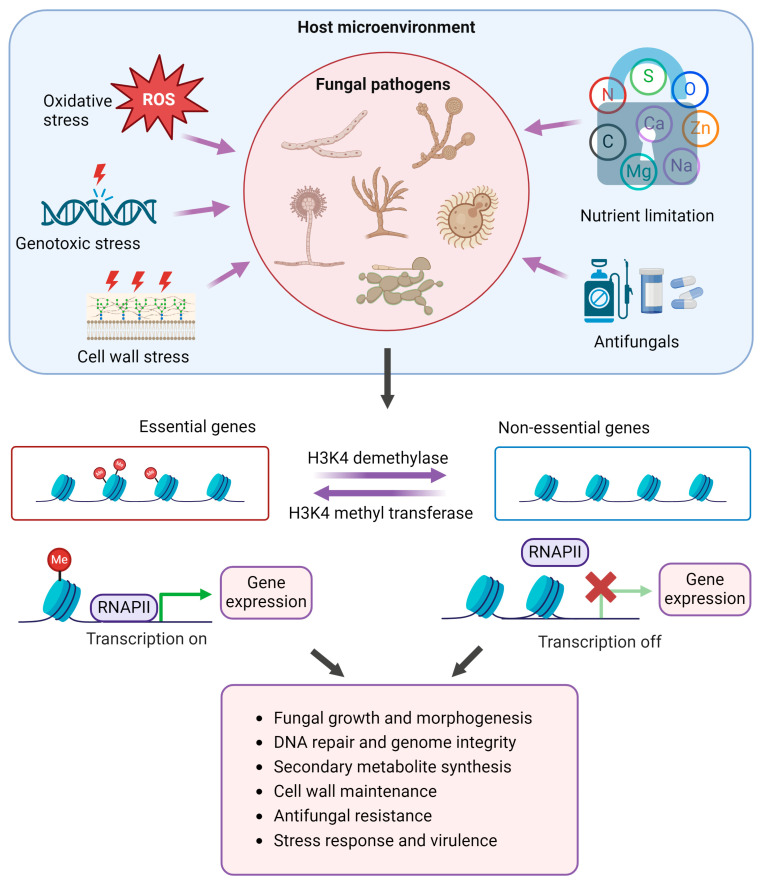
A graphical representation of the multifaceted roles of H3K4 methylation in the pathogenic adaptation of human and plant pathogenic fungi. Created in BioRender. Rai, N. (2024) https://BioRender.com/q87f061, 2 December 2024.

Epigenetic histone modifications are one of the main gene regulation mechanisms in eukaryotic organisms. Upon host invasion, fungal pathogens need to reorient their transcriptional machinery to cope with an adverse microenvironment and host immune response for their survival, adaptation, and proliferation. Post-translational histone modifications facilitate chromatin reorganization leading to the induction of desired gene expression to mount an appropriate response [11,72]. The contribution of histone modifications in fungal pathogenesis is still an evolving field and, so far, our understanding is limited on this topic (Figure 1). New emerging evidence strongly implicates the multifaceted roles of histone modification in the survival and proliferation of pathogenic fungi in their natural hosts. H3K4 methylation is commonly presumed to be a hallmark of active transcription in most genomic loci. It is not clear if H3K4 methylation is instructive for active transcription or plays a contributory role in active transcription; however, its association with active transcription is undeniable [34,44,55,73,74]. Nonetheless, in either case, it benefits fungal pathogens to mount appropriate transcriptional responses to environmental stresses [51,58,67,75]. H3K4 methylation modulates multiple cellular processes critical for fungal pathogenesis, such as growth and development, cell wall integrity, DNA repair, response to stress, metabolic remodeling antifungal resistance, and virulence (Figure 2).

The interplay between H3K4 methylation and gene regulation is complex and involves multiple transcription regulators, the assembly of complexes to modify histones, and other signal networks to achieve the desired results. Some reports in yeast and mammals suggest that H3K4me3 at promoter regions is often deposited in response to an increase in transcriptional activity and expands due to a feed-forward mechanism [76,77]. When located near the transcriptional start site (TSS), this modification promotes active transcription [76,77]. The mechanism of H3K4 methylation-mediated regulation appears to be partly common in some aspects and partly species-specific in others. For example, two phylogenetically closely related species, *S. cerevisiae* and *C. glabrata,* show elevated fluconazole resistance upon Set1 deletion, albeit employing unique mechanisms [66]. Similarly, some pathogens need H3K4 methylation for secondary metabolite production, while others shows higher secondary metabolites upon the loss of Set1 function [48,50,63,64].

Intriguingly, most of the reported roles of H3K4 methylation in fungal pathogens have been investigated by disrupting H3K4 methylation through gene deletion of histone methyl transferase (Set1/CclA) [48,51,58,64,75]. It will be interesting to find out how the loss of demethylases or a constitutive H3K4 methylation will affect the ability of fungal pathogens to adapt under different stress conditions. It is plausible that fungal cells when exposed to a host undertake both H3K4 methylation and demethylation at the same time but at different gene loci depending on the essentiality of the gene function under the given stress conditions (Figure 2). Examining the H3K4 methylation status of the fungal pathogens upon host infection or in vitro/in vivo infection models might shed light on the larger dynamic roles of H3K4 methylation in fungal pathogenesis. 

Another interesting question that remains to be answered is if H3K4 methylation can be a potential target to develop novel antifungal drugs. The roles of H3K9 and H3K36 methylation in antifungal drug resistance are explored but not for H3K4 methylation [78,79,80]. Importantly, epigenetic heterochromatin–gene silencing appear to contribute towards antifungal resistance in plant and human fungal pathogens [81]. Since SET1 regulates multiple processes pivotal in the pathogenesis of the human fungal pathogen *C. albicans*, and the Set1p N-terminal fragment does not show significant homology to other eukaryotic proteins, it may therefore be a novel potential therapeutic target [82]. Notably, a Jumonji histone demethylase inhibitor, JIB-04, showed antifungal activity against *Cryptococcus neoformans* by modulating ergosterol biosynthesis [83], suggesting that H3K4 methylation holds promise as an antifungal therapeutic target. Collectively, these studies underscore the significance of H3K4 methylation in the field of fungal pathogenesis, and suggest that targeting H3K4 methylation may pave the way for potentially novel and effective antifungal strategies in the future.
